# A Plea for the London Hospital

**Published:** 1888-06-16

**Authors:** Leopold de Rothschild, J. H. Buxton, F. C. Carr-Gomm


### A Plea for the London Hospital.

By H.R.H. the Duke of Cambridge, the Bishop of London, Cardinal Manning, and Messrs. Leopold de Rothschild, J. H. Buxton, and F. C. Carr-Gomm.

We would invoke your powerful assistance to direct public attention to the appeal which is now being made for the maintenance of the London Hospital.

Among the multitude of smaller, newer, and no doubt in many instances most deserving charities, which are now more or less urgently appealing to the charitable public, it is possible that the claims of our great East-end General Hospital may be overlooked or passed by.

The maintenance, however, of the London Hospital is—as H.R.H. the Prince of Wales said last year—of almost national importance. It is situated where it is most needed, in the heart of the vast East-end population—roughly estimated at upwards of a million and a quarter—consisting mostly of artizans, work people, dock and railway labourers, sailors, &c., to whom it is the only refuge in cases of accident or serious illness; but this situation which makes it so useful renders it at the same time unseen, and probably unheard of, by the wealthier portion of the metropolis.

During the year 1887 there were 8,863 In-patients treated within its walls. Of these 2,381 were accidents, 3,638 were extra urgent cases. There can be no doubt that the necessary closing of the Wards of other large general Hospitals has pushed a greater number than ever to "The London," where, though the assured income is small, and dependence is chiefly resting on voluntary contributions, the generosity of the public in past years has hitherto kept all the Wards open.

It is now only once every five years that an appeal for maintenance is made, and it is earnestly to be hoped that the response to the present appeal may be as sufficient as those to the appeals of 1878 and 1883. The average stay of an In-patient is nearly a month, at an average total cost of about five guineas, which for the 8,260 newly-admitted patients, makes a necessary expenditure of £43,365; the Out-Patients costing the comparatively small sum of £7,500. No effort is spared to confine the benefits of the Hospital to those for whom it is intended, viz., the poor, excluding both those who ought to pay for private medical attendance and those who should be dealt with by the parish authorities—except in cases of accident or urgency. The assured income is little over £16,000, and last year Hospital Sunday and Saturday Funds paid £3,333 and £575 respectively, but all these together go but two-fifths of the way to make up the required amount.

The Institution has during the last few years suffered the loss of some of its most generous supporters by death, and it is to be hoped that others will come forward to supply their place, and that many will send in their names to the Secretary, promising to subscribe something for the next five years.

It may be mentioned that during the last period (1883-1888) it was found necessary to provide better accommodation for the Nursing Staff; this has been done, and now every Nurse and Probationer has her own separate bedroom; but now no enlargement of the Hospital is either contemplated or would be desirable, and the pressing need for liberal help at the present time is merely to maintain this splendid old Hospital in working efficiency. All contributions should be sent to the Secretary, Mr. G. Q. Roberts, the London Hospital, Whitechapel, E.

